# Xylo-Oligosaccharide Production from Wheat Straw Xylan Catalyzed by a Thermotolerant Xylanase from Rumen Metagenome and Assessment of Their Probiotic Properties

**DOI:** 10.3390/microorganisms13112602

**Published:** 2025-11-15

**Authors:** Yajing Wu, Chanjuan Liu, Qinghua Qiu, Xianghui Zhao

**Affiliations:** Jiangxi Province Key Laboratory of Animal Nutrition, Engineering Research Center of Feed Development, Jiangxi Agricultural University, Nanchang 330045, China; wyj2711@163.com (Y.W.); chanjuanhx@163.com (C.L.); rcauqqh@cau.edu.cn (Q.Q.)

**Keywords:** rumen metagenomic xylanase, thermostable biocatalysis, lignocellulosic biomass conversion, xylo-oligosaccharides, prebiotic potential

## Abstract

A novel xylanase gene (*RuXyn854*) was identified from the rumen metagenome and was heterologously expressed in *Escherichia coli* to produce xylo-oligosaccharides (XOSs) as a prebiotic in this study. RuXyn854, a member of glycosyl hydrolase family 10, demonstrated peak enzymatic activity at pH 7.0 and 50 °C. RuXyn854 retains more than 50% of its activity after treatment at 100 °C for 10 min, highlighting the enzyme’s excellent heat resistance. RuXyn854 showed a preferential hydrolyzation of xylan, especially rice straw xylan. RuXyn854 activity was significantly increased in the presence of 15 mM Mn^2+^, 0.25% Tween-20, and 0.25% Triton X-100 (125%, 20%, and 26%, respectively). The reaction temperature (30, 40, and 50 °C), dosage (0.20, 0.27, and 0.34 U), and time (90, 120, and 150 min) of RuXyn854 affected the XOS yield and composition, with a higher yield at 0.27 U, 50 °C, and 120–150 min. Xylobiose, xylotriose, and xylotetraose were characterized as the predominant XOS products resulting from the enzymatic hydrolysis of wheat straw xylan by RuXyn854, with xylose present at a mere 0.49% of the total yield. The prebiotic potential of XOSs was assessed through in vitro fermentation with established probiotic strains of *Bifidobacterium bifidum* and *Lactobacillus brevis*. The results showed that, regardless of incubation time, XOSs stimulated the growth and xylanolytic enzyme secretion of the two probiotics compared to the controls. These results demonstrate that the feature of RuXyn854 to withstand temperatures up to 100 °C is impressive, and its ability to hydrolyze wheat xylan into XOSs promotes the growth of probiotics.

## 1. Introduction

Xylooligosaccharides (XOSs) are short-chain carbohydrates derived from xylan hydrolysis, composed of 2 to 10 xylose units linked through β-(1→4) glycosidic bonds [1]. XOS supplementation in animals has multifaceted prebiotic effects. XOSs promote the growth of beneficial gut bacteria, such as Bifidobacteria and Lactobacilli, leading to an improved gut microbiota composition and function [2]. This, in turn, enhances nutrient absorption and overall growth performance [3]. XOSs also support immune function by stimulating the production of short-chain fatty acids in the colon, providing an energy source for immune cells [4]. Additionally, XOSs can help reduce inflammation by curbing the production of pro-inflammatory cytokines and encouraging the release of anti-inflammatory factors [5]. These findings underscore the potential for XOSs as a functional feed additive or food for enhancing animal health and productivity and have been gaining significant attention.

Xylooligosaccharides (XOSs) are primarily prepared through enzymatic hydrolysis, chemical hydrolysis, and microbial fermentation [6,7]. Enzymatic hydrolysis uses specific enzymes, such as xylanases, to specifically break the β-(1→4) glycosidic bonds in xylan [6,8]. Chemical hydrolysis, such as acid and alkalis hydrolysis, when conducted under controlled conditions, can also produce XOSs from xylan, though it necessitates more expensive and robust equipment and may generate unwanted by-products, such as furfural, requiring further purification [6,9]. Microbial fermentation employs microorganisms like bacteria and fungi to act on xylan-rich substrates through their enzyme system [6,10]. However, this method has its limitations, including longer fermentation times, variable product yields due to the complexity of microbial metabolism, and potential additional purification steps to achieve high-purity XOSs. Therefore, enzymatic hydrolysis stands out as the preferred method for producing XOSs, with the selection of potent xylanases being key to achieving high yields, as well as to ensuring the quality and purity of the end product.

Xylanases sourced from diverse microbial origins, including fungi and bacteria, are leveraged in the production of XOSs from a range of lignocellulosic materials [11,12]. Despite this, the quest for ideal enzyme and substrate conditions for optimizing XOS yield is ongoing, highlighting the imperative for innovative xylanase identification [7]. Moreover, the potency of an XOS is shaped by its molecular weight distribution, contingent on the enzyme variant, the nature of the xylan starting material, preprocessing techniques, and the specific reaction conditions employed [7,13,14,15]. It is critical to select xylanases with low β-xylosidase activity to minimize xylose generation, which can detract from the efficacy of XOS synthesis [16].

The complex community of microorganisms in the rumen produces a variety of enzymes, including those that break down lignocellulose into fermentable sugars [17]. This natural repository of enzymes is key for finding effective xylanases for creating XOSs. Technological progress in DNA sequencing has made it possible to uncover and analyze these microbial genes more efficiently. The application of metagenomic techniques has led to the discovery of novel xylanase genes within the rumen [8,18,19]. Given the vast quantities of wheat straw produced each year—particularly in China—and its composition, rich in xylan, this by-product emerges as an ideal substrate for XOS production. In our study, we identified a specific xylanase gene (*RuXyn854*) from the rumen of cattle through genomic analysis. After cloning and expressing this gene, we assessed its ability to convert wheat straw-derived xylan into XOSs and studied the potential use of XOSs by beneficial microbes.

## 2. Materials and Methods

### 2.1. Gene Cloning and Expression Plasmid Construction

Our previous research provided a detailed understanding of the processes involved in DNA extraction from rumen microorganisms, followed by metagenomic sequencing and analysis [20]. The *RuXyn854* gene was synthesized using PCR with liquid-phase DNA as the template and the specific primers *RuXyn854F* (5′-AAGAAGGAGATATACGGATCCATGAAAAACAATCTTTTAATC-3′) and *RuXyn854R* (5′-GTGGTGGTGGTGGTGGTGCTCGAGGCGGGTCACGCGCACCTGAAT-3′). The PCR product was then cloned into the pET-28a vector using a one-step cloning kit (10911ES20; Yeasen Biotechnology (Shanghai) Co., Ltd., Shanghai, China), and the recombinant plasmid, named pET-RuXyn854, was transformed into *E. coli* DH5α cells. Positive clones were identified and sequenced. The sequence data has been deposited into GenBank (Accession: ON746678.1).

### 2.2. Sequence Analysis

The *RuXyn854* gene and its associated amino acid sequences were aligned using online tools provided by EBI (https://www.ebi.ac.uk/services, accessed on 11 March 2023). A phylogenetic tree was crafted for RuXyn854 using MEGA X version 10.2.6 software (https://www.megasoftware.net/, accessed on 11 March 2023), incorporating 50 analogous sequences from the alignment. Multiple sequence alignments were performed with ClustalW (https://www.genome.jp/tools-bin/clustalw, accessed on 11 March 2023), and InterPro was instrumental in identifying the conserved domains of RuXyn854. For homologous modeling, the Phyre2 server (http://www.sbg.bio.ic.ac.uk/phyre2/, accessed on 15 March 2023) was used, employing 3wp6 as a reference template.

### 2.3. Expression of RuXyn394

For the expression of RuXyn854, competent *E. coli* BL21(DE3) cells were transformed with the pET-RuXyn854 plasmid and selected on LB agar plates with kanamycin at 37 °C. PCR-verified transformants were cultured in LB liquid medium overnight before being transferred to fresh LB medium and grown to an OD_600_ of about 0.8. Expression was initiated with 0.4 mM IPTG at 20 °C, 80 rpm for 20 h, with non-transformed cells as a control. Cells were harvested by centrifugation, resuspended in PBS, and lysed by ultrasonication. The lysate was centrifuged, and the supernatant containing RuXyn854 was analyzed using SDS-PAGE and stained with Coomassie Blue. Purification was achieved using Ni-charged affinity chromatography; the supernatant was diluted with binding buffer (0.05 M sodium dihydrogen phosphate and 0.3 M sodium chloride) and passed through a 5 mL Ni-charged column. RuXyn854 was washed and eluted, and its concentration was determined using the Bradford assay. The purity of the protein was confirmed using SDS-PAGE. Additionally, Western blot analysis was performed using antibodies specific to the His-tag (66005-1-Ig, Proteintech Group, Inc., Chicago, IL, USA). It is noteworthy that the His-tag was positioned at the C-terminus of the RuXyn854 protein structure.

### 2.4. Characterization of RuXyn854

The substrate preferences of RuXyn854 were determined by assessing its activity on 1% of various xylan sources—wheat straw, rice straw, wheat bran, corn straw, and corn cob xylan, as well as chitosan and sodium carboxymethylcellulose (CMC-Na) or Avicel. Xylans were extracted from wheat straw, rice straw, corn straw, wheat bran, and corn cob following the methods outlined in a previous study [8]. These mixtures were incubated at 40 °C in a pH 6.0 buffer (0.05 M citric acid–disodium hydrogen phosphate) for 10 min. The amount of reducing sugars released was then quantified using the 3,5-dinitrosalicylic acid method, with absorbance readings taken at 540 nm to measure the colorimetric intensity.

The pH optimum for RuXyn854 activity was determined by testing its efficiency across a range of pH values, from 3.0 to 9.0, using a 0.05 M citric acid–disodium hydrogen phosphate buffer or Tris-HCl buffer, with 1% wheat straw xylan as the substrate at 40 °C. The enzyme’s temperature optimum was identified by evaluating its activity on wheat straw xylan at the determined pH optimum, with temperatures ranging from 20 to 100 °C. Activity data, normalized to a maximum of 100% relative activity, was used to illustrate the effects of pH and temperature. Additionally, the thermostability of RuXyn854 was assessed by pre-incubating the enzyme at temperatures between 20 and 100 °C for various durations (5, 10, 30, 60, 90, and 120 min) and then measuring the residual activity at the optimal pH and temperature conditions.

The specific activities of RuXyn854 were determined using 1% wheat straw xylan at 50 °C and pH 7.0 for 10 min. The enzymatic activity, defined as one unit (U), corresponded to the amount of enzyme capable of producing 1 μmol of reducing sugars per min. Specific activity was expressed as Units per milligram protein. In order to ascertain the *K*_m_, *V*_max_, and *k*_cat_ values of RuXyn854, a nonlinear regression analysis was employed, utilizing the Michaelis–Menten equation. The range of substrate concentrations spanned from 0.2% to 3.0%, featuring a variety of xylan sources. GraphPad Prism v5.0 (GraphPad Software Inc., San Diego, CA, USA) was utilized for this analytical process.

To evaluate the resilience of RuXyn854’s enzymatic activities against potential interferences from metal ions, inhibitors, and detergents, controlled amounts of these agents were independently incorporated into the enzymatic assay. The reactions were then carried out according to the established protocol. Activity measurements in the presence of these additives were compared against a control reaction, which was conducted without any additives and arbitrarily set at 100% activity to establish a reference point.

### 2.5. XOS Production and Assay

Our initial investigation focused on the impact of RuXyn854 dosage and temperature on the generation of XOSs from wheat straw xylan. We prepared reaction mixtures (1 mL) with varying amounts of RuXyn854 (0.20, 0.27, or 0.34 U) and a consistent 1% wheat straw xylan. These mixtures were incubated at pH 6.0, while the temperature was systematically altered to 30, 40, and 50 °C, with each condition maintained for 120 min. Subsequently, we explored the influence of temperature and reaction time on XOS yield. A standardized reaction mixture, containing 0.27 U of RuXyn854 and 1% wheat straw xylan, was subjected to the same pH and temperature variations but incubated for extended periods of 90, 120, and 150 min. Control reactions, containing only wheat straw xylan, were run under identical conditions to provide a baseline for comparison. The hydrolysis products (xylose, xylobiose, xylotriose, xylotetraose, and xylopentaose) were quantified using high-performance liquid chromatography (HPLC) with an Agilent Eclipse XDB-C18 column (4.6 × 250 mm, 5 μm), employing mannose as an internal standard according to our previous report. The term ‘total XOS’ refers to the combined quantity of xylobiose, xylotriose, xylotetraose, and xylopentaose. The amounts of individual XOSs and total XOSs released by RuXyn854 were calculated by subtracting the values obtained from the control group.

### 2.6. In Vitro Fermentation of XOS

*Levilactobacillus brevis* (CGMCC 1.2028) and *Bifidobacterium bifidum* (CGMCC 1.2212) were employed for in vitro fermentation of XOS. *L. brevis* was first cultured in medium A at 30 °C, which was composed of casein peptone (10 g/L), beef extract (10 g/L), yeast extract (5 g/L), glucose (5 g/L), sodium acetate (5 g/L), diamine citrate (2 g/L), Tween 80 (1 g/L), K_2_HPO_4_ (2 g/L), MgSO_4_·7H_2_O (0.2 g/L), and MnSO_4_·H_2_O (0.05 g/L). *B. bifidum* was cultured in medium B at 37 °C, containing soya peptone (5 g/L), tryptone (5 g/L), yeast extract (10 g/L), glucose (10 g/L), NaCl (40 mg/L), CaCl_2_ (8 mg/L), MgSO_4_·7H_2_O (19.2 mg/L), K_2_HPO_4_ (40 mg/L), KH_2_PO_4_ (40 mg/L), NaHCO_3_ (400 mg/L), L-cysteine (500 mg/L), and resazurin (1 mg/L). Crude XOSs were obtained by hydrolyzing wheat straw xylan with 0.27 U of RuXyn854 at 50 °C for 150 min, followed by filtration through a 0.22 μm membrane for sterilization and quantification using HPLC. The filtered XOSs are utilized for the subsequent probiotic fermentation process.

For the fermentation, overnight cultures of *L. brevis* and *B. bifidum* (10 μL each) were inoculated into 3 mL of glucose-free medium A or B, respectively, to which crude XOSs (1 mL) were subsequently added. Control groups received sodium acetate buffer with equivalent xylose and glucose concentrations. The bacteria were incubated at their respective optimal temperatures (30 °C for *L. brevis* and 37 °C for *B. bifidum*) for 24, 48, and 72 h. Post-incubation, growth metrics such as pH and OD_600_ were assessed. Enzymatic activity content in the supernatants was also analyzed.

### 2.7. Enzyme Assays

Enzymatic activities in the culture broths of *L. brevis* and *B. bifidum* were assessed according to the method described in our previous report [8]. Xylanase activity was quantified using a citric acid–disodium hydrogen phosphate buffer (pH 7.0) at 50 °C for 30 min, employing 1% wheat straw xylan as the substrate. The activity was defined in terms of the amount of enzyme necessary to release 1 μmol of xylose per minute under the given assay conditions. Additionally, β-xylosidase, α-galactosidase, β-galactosidase, and β-glucosidase activities were determined by monitoring the release of p-nitrophenol from the respective substrates: 4-nitrophenyl β-D-xylopyranoside, 4-nitrophenyl α-D-galactopyranoside, 4-nitrophenyl β-D-galactopyranoside, and 4-nitrophenyl β-D-glucopyranoside, respectively. The reaction mixture, consisting of the sample and 0.5 mL of 2 mM substrate, was incubated in a citric acid–disodium hydrogen phosphate buffer (pH 7.0) at 37 °C for 1 h. Similarly, one unit of these enzymatic activities was delineated as the quantity of enzyme required to release 1 μmol of *p*-nitrophenol per min under the assay conditions.

### 2.8. Statistical Analyses

The statistical analysis employed IBM SPSS statistics version 20 (IBM, Chicago, IL, USA). For comparisons involving three or more groups—including the effects of different temperature levels, various RuXyn854 dosages, and distinct incubation times on XOS synthesis (and composition), as well as the impacts of different XOS concentrations on the growth characteristics of *B. bifidum* and *L. brevis*—one-way analysis of variance (ANOVA) was applied. Before conducting multiple comparisons, we assessed the homogeneity of variances. Where significant heterogeneity was observed, Dunnett’s T3 post hoc test was applied for multiple comparisons. In cases where homogeneity was not significant, the Duncan test was utilized. For comparisons between two groups, an independent sample *t*-test was utilized. Statistical significance was established at *p* < 0.05. The results are presented as mean values with standard deviations (SD) in the accompanying figures and tables.

## 3. Results and Discussion

### 3.1. Sequence Analysis and Production of RuXyn854

**Figure 1:**
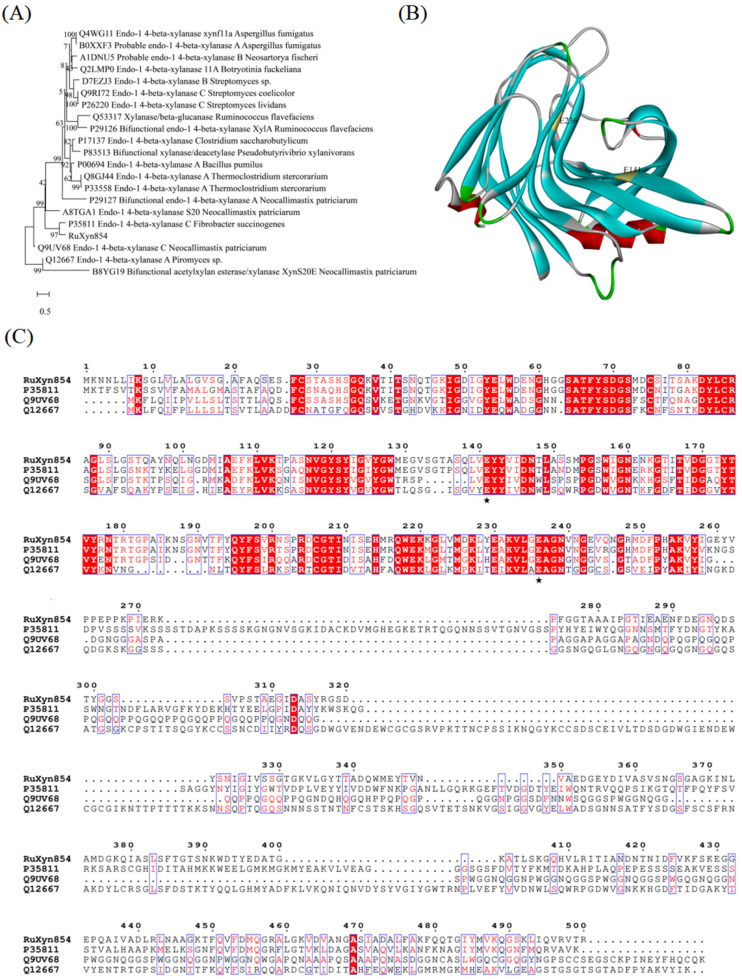
Phylogenetic tree (**A**), homology modeling (**B**), and multialignment analysis (**C**) of RuXyn854. Putative catalytic residues (pentacle).

The sequencing analysis reveals that the *RuXyn854* gene comprises 1503 base pairs, encoding a protein 500 amino acids in length. The theoretical molecular mass of this protein is estimated to be 53 kDa. The PSI-BLAST analysis, a tool for comparing protein sequences against a database to identify evolutionary relationships, was performed on EMBL-EBI’s platform (https://www.ebi.ac.uk/jdispatcher/sss/psiblast, accessed on 11 March 2023), utilizing sequences from the UniProtKB/Swiss-Prot Database with the default search parameters. This analysis indicated that RuXyn854 shares a high degree of sequence identity with several xylanases from *Fibrobacter succinogenes* (UniProtKB accession P35811), *Neocallimastix patriciarum* (UniProtKB accession Q9UV68), *Piromyces sp.* (UniProtKB accession Q12667), and *N. patriciarum* (UniProtKB accession A8TGA1), and within the GH11 family at the amino acid level. A phylogenetic tree, constructed by analyzing 20 protein sequences via PSI-BLAST, demonstrated a notable evolutionary relationship between RuXyn854 and the endo-1,4-beta-xylanases identified in *F. succinogenes* (UniProtKB accession P35811), as shown in Figure 1A. *F. succinogenes*, a key cellulolytic microorganism resident in the rumen, is renowned for its capacity to secrete a diverse repertoire of enzymes involved in cellulose degradation [21]. These include hemicellulases, cellulases, and carbohydrate esterases, which are essential for the efficient breakdown of fibrous substrates within the ruminal ecosystem. InterPro analysis precisely identified the GH11 domain within the RuXyn854 sequence, spanning amino acid residues 39 to 249, as illustrated in Supplementary Figure S1. Utilizing homology modeling, the three-dimensional structure of RuXyn394 was modeled with Phyre2, employing the crystal structure of 3wp6 as a reference template (Figure 1B). The predicted structure of RuXyn394 revealed a β-jelly-roll fold, akin to a partially closed hand, which is a distinctive feature of enzymes within the GH11 family. These results confirm that RuXyn854 belongs to the GH11 family. By aligning RuXyn854 with several other protein sequences known for their distinctive characteristics, we were able to discern the conserved patterns and specific elements within its sequence. Based on the aligned proteins, the catalytic sites are represented by two residues, E141 and E236, both indicated with a pentacle. Within the amino acid sequence from residues 1 to 260, RuXyn854 shows a high degree of sequence homology with the other compared sequences. However, between residues 260 to 500, the homology is significantly lower, which may be a key factor contributing to the unique characteristics of RuXyn854.

### 3.2. Expression and Characteristics of RuXyn854

**Figure 2:**
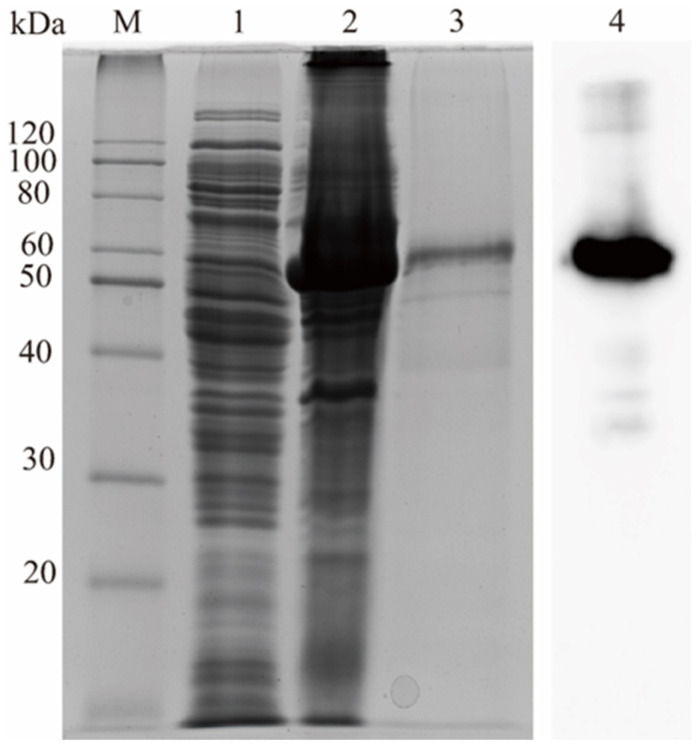
Analysis of RuXyn854 using SDS-PAGE and Western blotting. M, protein marker; 1, non-transformed *E. coli* BL21(DE3); 2, RuXyn854 transformants induced with 0.4 mM IPTG; 3, purified RuXyn854; 4, Western blot analysis of supernatant from the ultrasonication of RuXyn854 transformants.

The *RuXyn854* gene was heterologously expressed in *E. coli* BL21(DE3) cells. The subsequent SDS-PAGE analysis of the purified protein demonstrated a prominent band with an approximate molecular mass of 53 kDa, which is in concordance with the theoretical molecular weight predicted from the amino acid sequence of RuXyn854 (Figure 2). Western blot analysis also confirmed its identity as the RuXyn854 protein.

**Figure 3:**
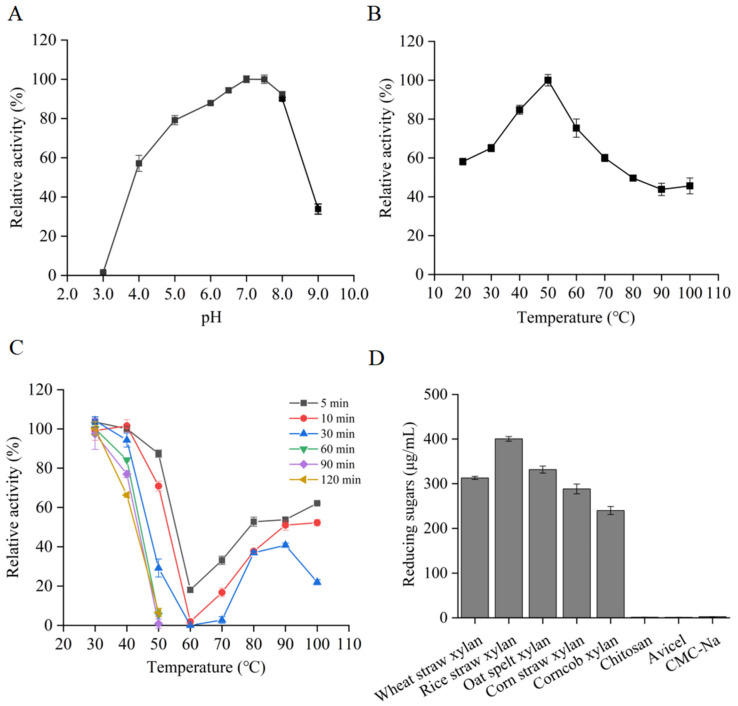
pH dependence (**A**), temperature dependence (**B**), thermostability (**C**), and substrate specificities (**D**) of RuXyn854.

The influence of pH and temperature on the activity of RuXyn854 was assessed using wheat xylan as the substrate. The enzyme demonstrated a broad pH adaptability, with its activity optimally peaking at pH 7.0 (Figure 3A), and maintained a high level of activity, exceeding 57%, within the pH range of 4.0 to 8.0. Additionally, RuXyn854 showed an exceptional temperature adaptability; its activity was highest at 50 °C and remained above 50% of the maximum throughout the temperature range of 20–80 °C. Remarkably, even at 100 °C, RuXyn854 retained 45% of its maximum activity (Figure 3B). RuXyn854 exhibits its highest activity at pH 7.0 and 50 °C, which is partially consistent with the rumen environment (generally 6.0–7.0 and 39–41 °C, respectively) and the results of some xylanases we previously reported [8,18]. For thermostability, RuXyn854 was pre-incubated at 30–100 °C for 5–120 min, and the residual activity was measured under optimal conditions. RuXyn854 shows no significant change in activity after treatment at 30 °C for 120 min, and it retains 66% of its maximum activity after treatment at 40 °C for the same duration, indicating that RuXyn857 has a good tolerance to both 30 and 40 °C (Figure 3C). If retaining over 50% of its maximum activity is considered the threshold, RuXyn854 can withstand 10 min at 50 °C, less than 5 min at 60 and 70 °C, 5 min at 80 °C, and 10 min at both 90 and 100 °C. This is an interesting finding, as it suggests that the thermal tolerance of RuXyn854 does not increase linearly with temperature. Different temperatures have unique impacts on RuXyn854. The tolerance of an enzyme to high temperatures is not necessarily inferior to its performance at low temperatures. Similarly to the current results, a xylanase from *Bacillus* sp. NTU-06 retains more activity after treatment at 40 °C compared to treatment at 10–30 °C [22]. RuXyn854 retains more than 50% of its activity after treatment at 100 °C for 10 min, highlighting the enzyme’s excellent heat resistance, a characteristic that many other enzymes cannot match (Table 1). Similarly to the current study, a xylanase named rMxyl from soil was found to retain 50% and over 40% of its activity after being treated at 90 °C for 15 and 30 min, respectively [23] (Table 1). However, it is regrettable that the study did not report the performance of rMxyl after treatment at 100 °C [23].

To evaluate the substrate specificity of RuXyn854, a range of substrates including chitosan, microcrystalline cellulose, sodium carboxymethyl cellulose (CMC-Na), and four distinct xylans were utilized. The results demonstrated a significant preference of RuXyn854 for degrading xylan, with negligible activity observed towards the other substrates, confirming its typical characteristics as a xylanase (Figure 3D). Among the tested xylans, the enzyme exhibited the highest activity towards rice straw xylan, followed by wheat straw xylan, and the lowest towards corn cob xylan. RuXyn854 exhibits significant differences in catalytic efficiency when acting on various xylans as substrates. Specifically, the enzyme demonstrates the highest catalytic constants (*k*_cat_), catalytic efficiency (*k*_cat_/*K*_m_), and specific activity when rice straw xylan is the substrate (Table 2). In contrast, these activity parameters are at their lowest when corn cob xylan is utilized. This trend is consistent with previous findings regarding substrate specificity. This variability in activity may correlate with differences in the chemical composition of the xylans derived from various sources. Similar results were observed in our previous study [8].

**Figure 4:**
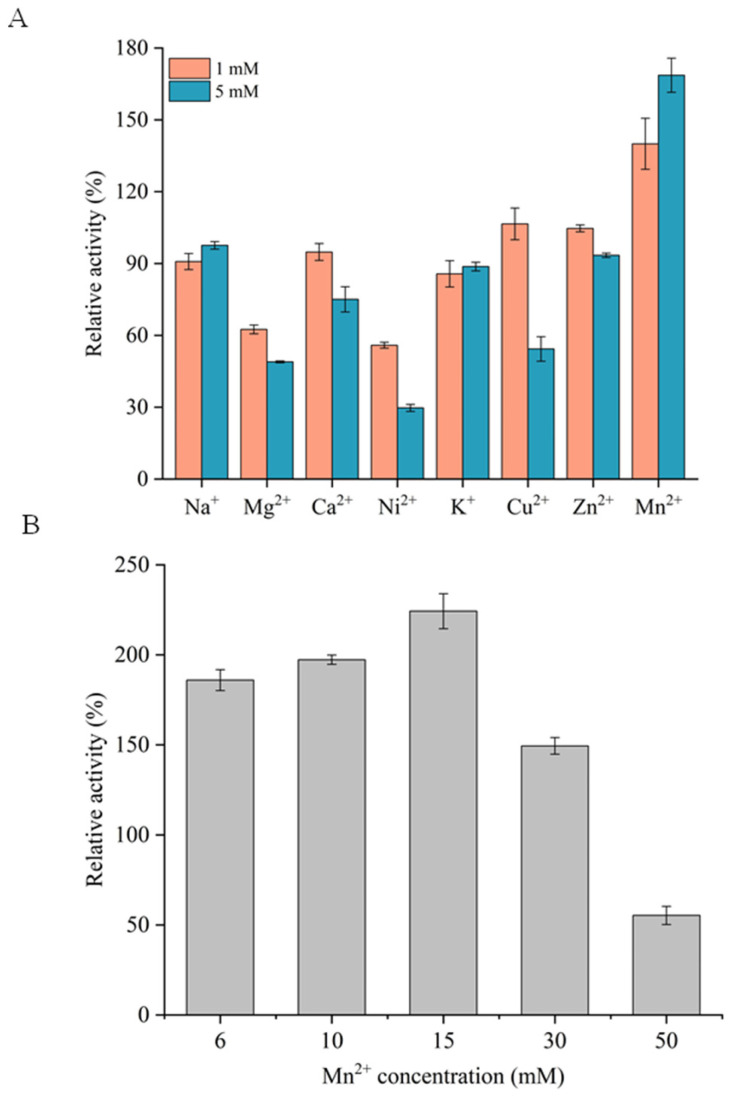
Effects of various metal ions (**A**) and Mn^2+^ concentration (**B**) on RuXyn854’s activity.

This study investigated the effects of eight metal ions, namely Na^+^, Mg^2+^, Ca^2+^, Ni^2+^, K^+^, Cu^2+^, Mn^2+^, and Zn^2+^, on the activity of RuXyn854 (Figure 4A). When the concentration of these ions was 1 mM, the activity of RuXyn854 remained above 56% of its maximum activity. Upon increasing the ion concentration to 5 mM, with the exception of Mg^2+^ and Ni^2+^, which inhibited 51.1% and 70.3% of RuXyn854’s activity, respectively, the remaining ions still maintained over 50% of the enzyme’s activity. These data indicate that RuXyn854 is resistant to the majority of ions tested in this study. Notably, its resistance to Cu^2+^ sets it apart from many rumen-derived xylanases reported in the literature [8,18,30], where 5 mM Cu^2+^ nearly inactivated the xylanases in those studies. Additionally, Mn^2+^ exerts a unique activating effect on RuXyn854, with 1 mM and 5 mM concentrations of Cu^2+^ enhancing its activity by 40.0% and 68.6%, respectively. Upon further investigation with Mn^2+^ concentrations elevated to 50 mM, we observed that RuXyn854 activity initially increased and subsequently decreased with rising Mn^2+^ levels, reaching a peak stimulation at 15 mM Mn^2+^, which amplified RuXyn854 activity by 125.2% (Figure 4B). The augmenting effect of Mn^2+^ on the activity of xylanases has been confirmed in various studies [18,24,30,31]. This suggests that Mn^2+^ may be critically important for enzymatic activity, potentially through interactions with key amino acid residues at the enzyme’s active site, serving as a cofactor, or by causing conformational changes in the enzyme’s tertiary structure [30].

The influence of various inhibitors and organic solvents on the activity of RuXyn854 is presented in Table 3. RuXyn854 demonstrates a significant resistance to SDS, dithiothreitol, EDTA-Na, β-mercaptoethanol, Tween-20, and Triton X-100, maintaining over 64.3% of its activity even at relatively high concentrations of these reagents. Notably, RuXyn854’s performance in the presence of 5 mM SDS is superior to that of many xylanases previously reported [8,32], which are prone to inactivation under the same conditions. This suggests that RuXyn854 may hold considerable potential for applications in the textile industry [33]. The current findings are akin to those of a GH11 xylanase we have previously reported, which retained 68% of its activity at 5 mM SDS [18]. The prediction found that RuXyn854 does not contain any disulfide bonds, which might be the reason why dithiothreitol and β-mercaptoethanol have little impact on its activity [34]. The suppression of RuXyn854 activity upon interaction with EDTA, an agent that sequesters metals, implies a necessity for metal ions in the enzyme’s catalytic process [35]. This is further confirmed by the observed increase in RuXyn854 activity upon the addition of Mn^2+^. The activity of RuXyn854 was enhanced by 19.7% and 25.5%, respectively, upon the addition of 0.25% Tween-20 and Triton X-100. Enhanced xylanase activity in response to Tween-20 and Triton X-100 has been documented in both *Aspergillus japonicus* [36] and rumen-derived xylanases from our earlier work [8,20]. Acting as nonionic surfactants, these compounds facilitate enzymatic hydrolysis through several mechanisms: they encourage the dispersion of proteins to reveal active sites, alter the interaction between enzymes and their substrates, and amplify the enzymes’ catalytic action [37,38]. The current findings indicate that the residual activity of RuXyn854 remains above 64.7% when the reaction system contains 10% methanol, ethanol, and dimethyl sulfoxide (DMSO). However, when the concentration of these organic solvents is elevated to 30%, the residual activity of RuXyn854 drops to an average of 24.3%. These results suggest that RuXyn854 possesses a notable tolerance to the effects of organic solvents at certain concentrations.

### 3.3. Production and Assay of XOS

**Figure 5:**
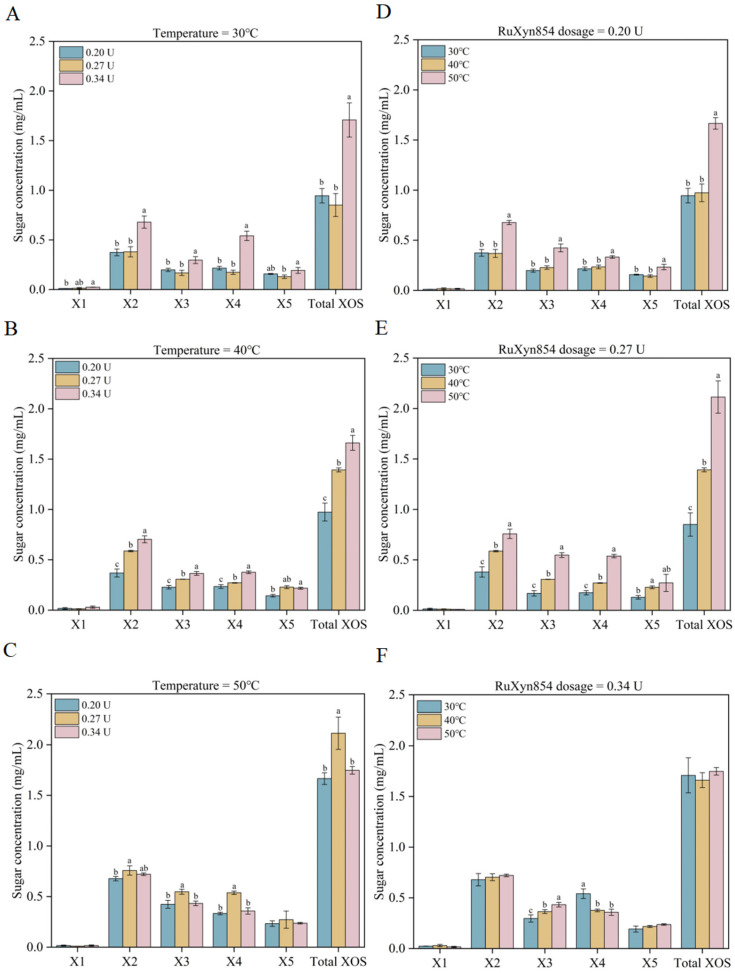
XOS synthesis under varied temperature conditions and RuXyn854 dosages. (**A**–**C**), the impact of RuXyn854 dosages on XOS yield at constant incubation temperatures of 30 °C, 40 °C, and 50 °C, respectively. (**D**–**F**), the influence of incubation temperature on XOS synthesis when RuXyn854 is applied at dosages of 0.20 U, 0.27 U, and 0.34 U, respectively. The term ‘Total XOS’ refers to the cumulative quantity of xylobiose, xylotriose, xylotetraose, and xylopentaose. Within each category (individual XOSs or Total XOSs), values marked with distinct letters are statistically distinct at the 0.05 level of significance.

The effects of reaction temperature and enzyme dosage on the formation of xylooligosaccharides (XOSs) from wheat straw xylan were investigated in this study. At 30 °C, a RuXyn854 dosage from 0.20 U to 0.27 U did not significantly alter the yields of xylose, xylobiose, xylotriose, xylotetrose, xylopentaose, or total XOSs (Figure 5). However, when the dosage of RuXyn854 was increased to 0.34 U, the yields of xylobiose, xylotriose, xylotetrose, and total XOSs were significantly enhanced, reaching 0.68, 0.30, 0.54, and 1.71 mg/mL, respectively. At 40 °C, the yields of xylobiose, xylotriose, xylotetrose, and total XOSs significantly increased with the increase in RuXyn854 dosage. Yet, at 50 °C, the 0.27 U group exhibited higher yields of xylobiose, xylotriose, xylotetrose, and total XOSs compared to the other two dosage groups. When the RuXyn854 dosage was 0.20 U and 0.27 U, the yields of xylobiose, xylotriose, xylotetrose, xylopentaose, and total XOSs reached their peak as the reaction temperature increased from 30 °C to 50 °C, particularly under the combination of 50 °C and 0.27 U, where the yields were 0.76, 0.55, 0.54, 0.27, and 2.11 mg/mL, respectively. This may be related to the optimal reaction temperature for RuXyn854 being 50 °C. However, at a RuXyn854 dosage of 0.34 U, although the yield of xylotriose increased with the temperature rise from 30 °C to 50 °C, the yield of xylotetrose decreased, resulting in no significant difference in total XOS yields among the temperature groups. The results indicate an interactive effect between the reaction temperature and enzyme dosage, where the highest reaction temperature and enzyme dosage do not always lead to the maximum yield of XOSs. Similar results have also been observed in previous studies [39]. High enzyme dosages at elevated temperatures did not lead to an increased production of XOSs, which may be associated with the aggregation of enzymes under high enzymatic loads during the incubation period [40].

**Figure 6:**
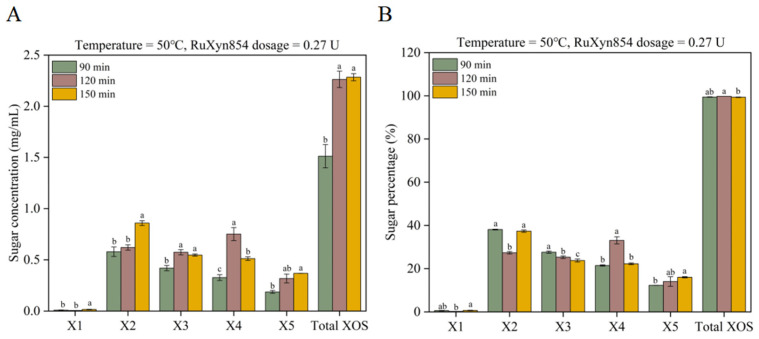
The impact of incubation time on XOS synthesis (**A**) and composition (**B**). Within each category (individual XOSs or Total XOSs), values marked with distinct letters are statistically distinct at the 0.05 level of significance.

We further explored the impact of duration time on XOS yield, selecting the more favorable temperature (50 °C) and dosage (0.27 U) from the previous experiment for use in subsequent trials. When the reaction time was extended from 90 to 120 min, the yields of xylose, xylobiose, and xylopentaose remained unchanged, while the yields of xylotriose, xylotetrose, and total XOSs significantly increased (Figure 6A). Prolonging the reaction time further to 150 min resulted in a significant increase in the yields of xylose and xylobiose, a significant decrease in xylotetrose, with no significant changes observed for xylotriose or total XOSs. In terms of the sugar composition in the degradation products, different reaction durations led to distinct characteristics (Figure 6B). Compared to the reaction times of 90 and 150 min, the 120 min duration with RuXyn854 produced a lower proportion of xylose and xylobiose, a higher proportion of xylotetrose and total XOSs, with the proportions of xylotriose and xylopentaose falling in between. Specifically, the proportions of xylobiose and xylotetrose decreased and increased by 10.4 and 11.2 percentage points, respectively. Compared to the 90 min reaction time, the 150 min duration did not significantly differ in the proportions of xylose, xylobiose, xylotriose, and total XOSs, but there was a decrease and increase of approximately 3.8 percentage points for xylotriose and xylopentaose, respectively. In summary, across the three distinct reaction durations, xylose constituted an average of only 0.49% of the products, suggesting that the total proportion of XOSs averaged at 99.51%. This indicates that the enzyme RuXyn854 is highly suitable for the production of XOSs. In the production process of XOSs, the generation of xylose is typically not desired, as it can inhibit the continued production of XOSs [40]. Furthermore, XOSs with a degree of polymerization (DP) of 2–4 are the preferred types of oligomers [41], especially in the application of the food industry, because XOSs with a shorter average DP exhibit better fermentation kinetics when producing value-added products [40,42,43]. In the current study, the yield of xylopentaose was lower than that of other oligosaccharides, which further confirms the greater potential for application of XOSs produced by RuXyn854 in the food industry.

### 3.4. In Vitro Fermentation of XOSs

Subsequently, the impact of XOSs on the growth characteristics of probiotics in the gut was explored, taking *B. bifidum* and *L. brevis* as examples (Table 4). Compared with the control group, XOSs significantly increased the OD_600_ values of *B. bifidum* after 24 and 48 h of cultivation. Additionally, regardless of the cultivation duration, XOSs markedly enhanced the OD_600_ values of *L. brevis*. These findings indicate that both *B. bifidum* and *L. brevis* are capable of utilizing XOSs to promote their growth. These findings are consistent with numerous previous reports [8,44,45]. XOSs undergo metabolism within probiotic bacteria through the concerted action of a variety of specific proteins, including enzymes that degrade and metabolize XOSs, transport proteins, and regulatory proteins [41,46]. Regardless of the cultivation duration, XOSs significantly enhanced the activities of various enzymes in the fermentation broth of *B. bifidum* and *L. brevis*, including xylanase, β-glucosidase, β-galactosidase, α-galactosidase, and β-xylosidase. This phenomenon is not only associated with the growth and proliferation of both strains promoted by XOSs but may also be related to the inductive effect of XOSs on the activity of these enzymes. This is especially evident with respect to the β-xylosidase enzyme from *L. brevis*, where no activity was observed in the control group during the 48 h fermentation period. However, the XOS group exhibited a significant β-xylosidase activity level of 147.4 U/L. These findings also substantiate the critical role of β-xylosidase in the probiotic metabolism of XOSs, a function that is central to the enzyme’s breakdown of XOSs into xylose and has been underscored in the extant literature [41,46]. Corroborating our results, Iliev et al. (2020) observed that XOSs induced the intracellular synthesis of β-xylosidase, β-glucosidase, and xylanase in *L. plantarum*, *L. brevis*, and *L. sake* when compared to glucose as a substrate [46]. However, in contrast to their study, our research detected these enzymes in an extracellular state, a finding that is congruent with additional reports indicating a significant augmentation of these enzymes’ activities in the fermentation medium of probiotics by XOSs [8,47]. For the strains *B. bifidum* and *L. brevis*, certain detection indices were influenced by the incubation time in both the control and XOS-treated groups, yet the impact did not follow a discernible pattern. Taking *B. bifidum* as an example, the OD_600_ in the XOS group significantly decreased with extended incubation time, while the xylanase activity paradoxically increased. Conversely, the activities of α-galactosidase and β-xylosidase exhibited an opposing trend, rising markedly with prolonged cultivation. Similarly, the OD_600_ of *L. brevis* increased with incubation time, accompanied by a corresponding rise in the activities of β-galactosidase and β-xylosidase. In contrast, xylanase activity significantly declined, and the activity of α-galactosidase exhibited a biphasic pattern, initially increasing before subsequently decreasing. It is relatively straightforward to comprehend the indices that align with the OD_600_ variation trends of *B. bifidum* and *L. brevis*. However, the reasons behind the inconsistencies in other indices remain elusive and challenging to elucidate.

## 4. Conclusions

In the current investigation, a novel xylanase gene, designated *RuXyn854*, belonging to GH11, was identified from a rumen metagenome and successfully heterologously expressed in *E. coli*. This enzyme displayed an optimal activity at pH 7.0 and 50 °C, coupled with a broad operational temperature spectrum and remarkable thermal stability within the range of 90 to 100 °C. RuXyn854 was significantly activated by Mn^2+^ concentrations less than 15 mM and exhibited a tolerance to 5 mM SDS as well as to 10% ethanol and methanol. The reaction temperature, dosage, and time of RuXyn854 affected the yield and composition of XOSs, with a higher yield obtained at a dosage of 0.27 U, reaction temperature of 50 °C, and reaction time of 120 and 150 min. In the hydrolysis products of wheat straw xylan with RuXyn854, only trace amounts of xylose were detected, while xylobiose, xylotriose, and xylotetrose were identified as the primary XOS products. XOS supplementation stimulated the growth and xylanolytic enzyme secretion of *B. bifidum* and *L. brevis* in vitro. These results demonstrate that the feature of RuXyn854 to withstand temperatures up to 100 °C is impressive, and its ability to hydrolyze wheat xylan into XOSs promotes the growth of probiotics.

## Figures and Tables

**Table 1:** Comparison of RuXyn854 with other GH11 xylanases.

Xylanase (Source)	Molecular Weight (kDa)	T_opt,_ pH_opt_	Thermostability (% Residual Activity, Maximum Temperature, Time)	Kinetic Values (Temperature, Substrate)	Hydrolysis Products (Substrate)	Ref.
CrXyn (rumen metagenome)	38	40 °C, 7.0	Inactivation, 70 °C, 30 min	*K*_m_ 5.98 g/L (40 °C, wheat straw xylan)	X1 (10.5%), X2 (58.1%), X3 (21.0%), X4 (5.3%), X5 (5.2%) from wheat straw xylan	[18]
XylCMS (rumen)	47	55 °C, 6.0	>80%, 60 °C, 10 min	*K*_m_ 23.3 g/L, *k*_cat_ 1383 s^−1^ (55 °C, oat spelt xylan)	X4 from oat spelt xylan	[19]
XynNTU (*Paenibacillus campinasensis*)	41	60 °C, 7.0	<10%, 80 °C, 3 h	*K*_m_ 8.35 g/L (60 °C, oat spelt xylan)	X2, X3, X5 from beech-wood xylan	[24]
rMxyl (compost–soil metagenome)	40	80 °C, 9.0	50%, 90 °C, 15 min	*K*_m_ 8.0 g/L (80 °C, birchwood xylan)	X1, X2, X3 from wheat bran	[23]
Xyn11-1 (saline–alkali soil)	27	50 °C, 6.0	23.7%, 50 °C, 5 min	*K*_m_ 3.7 g/L, *k*_cat_ 42.1 s^−1^ (50 °C, birchwood xylan)	X4, X5 from birchwood xylan	[25]
PphXyn11 (*Paenibacillus physcomitrellae*)	20.2	40 °C, 3.0–4.0	Near-inactivation, 50–80 °C, 1 h	*K*_m_ 13.8 g/L, 149.34 s^−1^ (40 °C, birchwood xylan)	X2, X3 from X4, X5, and X6	[26]
xylanase (*Aspergillus tamari*)	19.5	60 °C, 5.5	Inactivation, 60 °C, 30 min	*K*_m_ 7.9 g/L, 408.2 s^−1^ (60 °C, birchwood xylan)	X2, X3, and X4 from birchwood xylan	[27]
Thxyn11A (*Thermobifida halotolerans*)	34	70 °C, 9.0	<10%, 90 °C, 30 min	*K*_m_ 3.5 g/L (70 °C, birchwood xylan)	X3, X4, X5 from birchwood xylan	[28]
Bpu XynA (*Bacillus pumilus*)	23	50 °C, 6.6	>80%, 65 °C, 10 min	*K*_m_ 5.53 g/L, 56.07 s^−1^ (60 °C, oat spelt xylan)	X1 (trace amount), X2, X3, X4 from oat spelt xylan	[29]
RuXyn854 (rumen metagenome)	53	50 °C, 7.0	52%, 100 °C, 10 min	*K*_m_ 150.1 g/L, 416.1 s^−1^ (50 °C, wheat straw xylan)	X1 (trace amount), X2, X3, X4, X5 from wheat straw xylan	This study

**Table 2:** Kinetic parameters of RuXyn854.

Substrates	*K*_m_ (mg mL^−1^)	*V*_max_ (μmol min^−1^ mg^−1^)	*k*_cat_ (s^−1^)	*k*_cat_/*K*_m_ (mL mg^−1^ s^−1^)	Specific Activity (U/mg)
Rice straw xylan	103.3	559.1	493.9	4.78	353.5
Wheat straw xylan	150.1	471.0	416.1	2.77	276.9
Corn straw xylan	150.0	449.6	397.2	2.65	252.2
Corn cob xylan	49.7	119.0	105.1	2.12	208.8

**Table 3:** Effects of various regents on RuXyn854’s activity (%).

Reagents	Concentration	Relative Activity (%)
SDS	1 mM	99.0 ± 1.2
	5 mM	75.2 ± 3.0
Dithiothreitol	1 mM	111.7 ± 5.2
	5 mM	111.7 ± 1.2
EDTA-Na	1 mM	58.8 ± 3.5
	5 mM	64.3 ± 6.1
β-mercaptoethanol	1 mM	100.2 ± 3.4
	5 mM	97.5 ± 1.7
Tween-20	0.05% (*v*/*v*)	115.3 ± 5.5
	0.25% (*v*/*v*)	119.7 ± 1.8
Triton X-100	0.05% (*v*/*v*)	117.7 ± 2.6
	0.25% (*v*/*v*)	125.5 ± 8.7
Methanol	5% (*v*/*v*)	83.9 ± 3.7
	10% (*v*/*v*)	64.7 ± 4.7
	30% (*v*/*v*)	23.6 ± 0.8
Ethanol	5% (*v*/*v*)	77.3 ± 4.7
	10% (*v*/*v*)	71.3 ± 4.9
	30% (*v*/*v*)	26.7 ± 0.9
DMSO	5% (*v*/*v*)	51.1 ± 0.9
	10% (*v*/*v*)	69.8 ± 2.6
	30% (*v*/*v*)	22.5 ± 3.0

**Table 4:** Effects of XOSs on growth characteristics of *B. bifidum* and *L. brevis* with enzyme activity.

Item	Time (h)	*B. bifidum*		*p* Value	*L. brevis*		*p* Value
		Control	XOS		Control	XOS	
OD_600_	24	1.43 ± 0.22	1.84 ± 0.07 ^a^	0.012	0.84 ± 0.42 ^b^	1.87 ± 0.22 ^b^	<0.01
	48	0.93 ± 0.29	1.57 ± 0.19 ^b^	0.010	1.38 ± 0.52 ^ab^	2.31 ± 0.09 ^a^	0.037
	72	0.94 ± 0.60	1.30 ± 0.09 ^c^	0.277	2.00 ± 0.14 ^a^	2.43 ± 0.11 ^a^	<0.01
pH	24	6.88 ± 0.04	6.84 ± 0.04	0.193	6.93 ± 0.06 ^c^	6.86 ± 0.17 ^c^	0.494
	48	6.99 ± 0.06	6.81 ± 0.16	0.083	7.39 ± 0.20 ^b^	7.30 ± 0.24 ^b^	0.572
	72	7.02 ± 0.13	6.74 ± 0.09	0.012	7.68 ± 0.20 ^a^	7.72 ± 0.11 ^a^	0.772
Xylanase (U/L)	24	82.16 ± 2.99	222.56 ± 9.51 ^a^	<0.01	84.41 ± 5.31	171.44 ± 16.40 ^a^	<0.01
	48	76.97 ± 7.88	213.31 ± 16.80 ^a^	<0.01	89.11 ± 11.78	156.75 ± 15.26 ^ab^	<0.01
	72	79.94 ± 8.66	150.53 ± 9.38 ^b^	<0.01	110.63 ± 17.21	135.92 ± 9.51 ^b^	0.090
β-glucosidase (U/L)	24	47.66 ± 2.66 ^b^	85.72 ± 2.14	<0.01	34.13 ± 0.93 ^c^	81.79 ± 17.72	0.012
	48	62.31 ± 8.16 ^a^	91.61 ± 5.11	<0.01	37.01 ± 1.19 ^b^	92.80 ± 19.79	<0.01
	72	49.90 ± 1.57 ^b^	91.19 ± 6.55	<0.01	44.02 ± 3.05 ^a^	86.77 ± 6.78	<0.01
β-galactosidase (U/L)	24	39.32 ± 0.70 ^b^	173.47 ± 5.08	<0.01	31.19 ± 2.94	219.17 ± 9.78 ^b^	<0.01
	48	41.70 ± 2.26 ^ab^	147.82 ± 30.92	<0.01	42.75 ± 13.24	229.61 ± 12.38 ^ab^	<0.01
	72	43.88 ± 2.27 ^a^	151.88 ± 33.88	<0.01	42.05 ± 7.21	246.01 ± 12.11 ^a^	<0.01
α-galactosidase (U/L)	24	40.93 ± 1.82	74.01 ± 1.35 ^b^	<0.01	33.85 ± 1.28	75.56 ± 4.30 ^b^	<0.01
	48	42.82 ± 2.94	78.08 ± 4.45 ^ab^	<0.01	35.32 ± 1.87	147.82 ± 5.61 ^a^	<0.01
	72	42.75 ± 1.13	82.70 ± 3.92 ^a^	<0.01	43.88 ± 9.86	90.38 ± 7.96 ^c^	<0.01
β-xylosidase (U/L)	24	9.53 ± 1.19	41.63 ± 1.16 ^b^	<0.01	ND ^b^	50.74 ± 8.00 ^b^	<0.01
	48	12.06 ± 2.42	54.67 ± 11.73 ^b^	<0.01	ND ^b^	147.40 ± 14.93 ^a^	<0.01
	72	12.48 ± 1.42	92.10 ± 22.62 ^a^	<0.01	4.14 ± 0.95 ^a^	135.55 ± 10.14 ^a^	<0.01

Within the same metrics, means within same column with no common letters differ significantly (*p* ≤ 0.05). ND, non-detected.

## Data Availability

The original contributions presented in this study are included in the article/Supplementary Materials. Further inquiries can be directed to the corresponding author.

## Supplementary Materials

The following supporting information can be downloaded at https://www.mdpi.com/article/10.3390/microorganisms13112602/s1. Figure S1: InterPro analysis of RuXyn854.
