# Supplementary figures and images for: Xylo-Oligosaccharide Production from Wheat Straw Xylan Catalyzed by a Thermotolerant Xylanase from Rumen Metagenome and Assessment of Their Probiotic Properties
Source: Microorganisms. 2025 Nov 15;13(11):2602. doi: 10.3390/microorganisms13112602 (PMC12654769; doi:10.3390/microorganisms13112602)

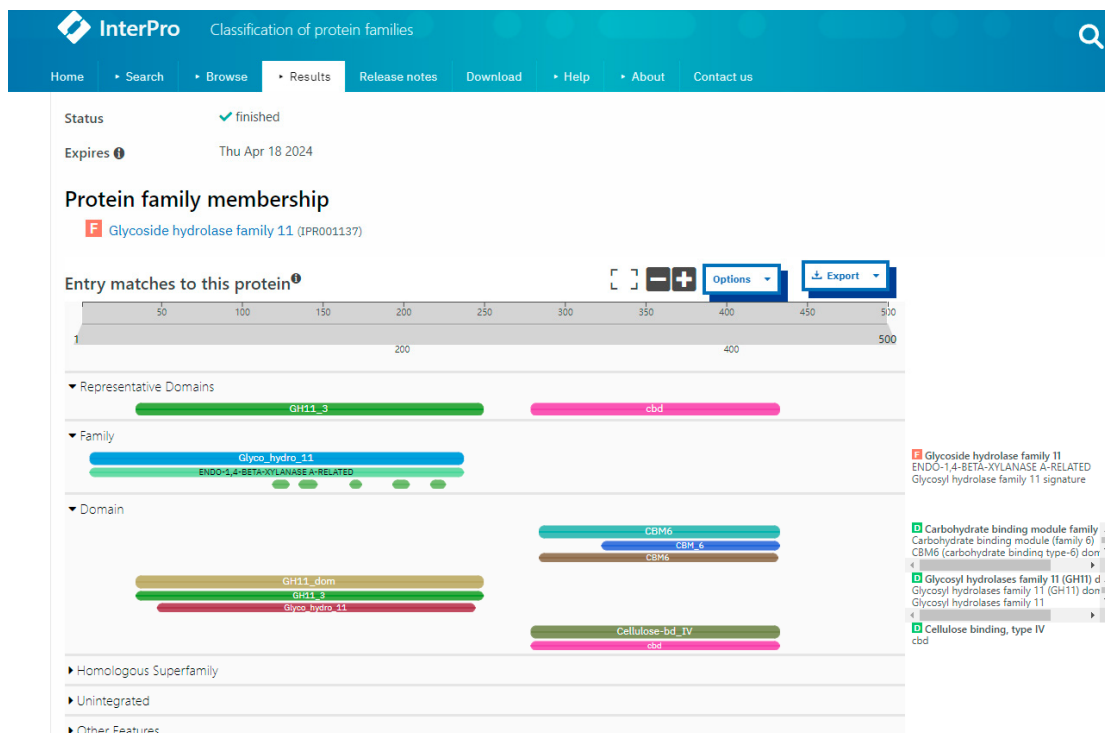

**Figure S1.** InterPro analysis of RuXyn854.

Supplement: Supplementary file 1 [file microorganisms-13-02602-s001.zip › microorganisms-3943638-supplementary.pdf]
